# Hepato-Protective Effect of Pomegranate and Persimmon Juices Against Oxidative Stress and Inflammation in Cyclosporine-Induced Cholestasis in Rats

**DOI:** 10.3390/foods15091473

**Published:** 2026-04-23

**Authors:** Rasha S. Mohamed, Karem Fouda

**Affiliations:** Nutrition and Food Sciences Department, National Research Centre, Cairo 12622, Egypt; karemfouda@gmail.com

**Keywords:** cholestasis, cyclosporine, pomegranate juice, persimmon juice

## Abstract

Background: Oxidative liver damage, fibrosis, cirrhosis and liver failure are caused by reactive oxygen species and inflammatory responses triggered by bile retention during prolonged cholestasis. Pomegranate and persimmon fruits, which are loaded with bioactive compounds that have anti-inflammatory and antioxidant properties, were evaluated separately for their efficacy in preventing oxidative stress and inflammation in cholestasis. Methods: Pomegranate and persimmon juices were analyzed for their vitamin C, carotenoids and organic acid levels, phenolic profile, and antioxidant activity. Liver protection against oxidative stress and inflammation brought on by cyclosporine-induced cholestasis in rats was verified by biochemical measurements, metabolite identification, and histopathologic examination. To forecast the mechanism of pomegranate and persimmon anti-inflammatory action, an in silico assessment was also carried out. Results: Vitamin C levels in pomegranate and persimmon juices were 99.55 and 51.75 µg/g, respectively. In both pomegranate and persimmon juices, gallic acid was the most prevalent phenolic compound (123.20 and 50.69 µg/g, respectively). Pomegranate and persimmon juices significantly (*p* < 0.05) reduced the rise in liver values of MDA, NO, TNF-α, IL-6, IL-1β, and TLR4, as well as serum values of total and direct bilirubin caused by cyclosporine. Additionally, the alteration of metabolites, particularly amino acids, demonstrated the inhibitory effect of pomegranate and persimmon juices on liver damage. Gallic acid’s and catechin’s substantial binding affinities with target inflammatory cytokines (TNF-α and TLR4) were further validated by molecular docking. Conclusions: These results showed that pomegranate and persimmon juices mainly modulated inflammation and oxidative stress to provide hepato-protective benefits against cyclosporine-induced cholestatic liver injury.

## 1. Introduction

The liver is an integral part of bile anabolism and detoxification as well as regulating bile secretion [1]. The clinical signs of cholestasis, a prevalent liver disorder in clinics, include excessive bile component accumulation and obstruction of bile flow [2]. According to Zhang et al. [3], chronic and persistent cholestasis might eventually result in liver fibrosis, cirrhosis, an elevated risk of cholangiocarcinoma, hepatocellular carcinoma, and liver failure, which in extreme circumstances can be lethal. After bile duct ligation, rats’ bile acid concentrations rise, which causes lipid peroxidation. This is most likely caused by the enhancement of phagocyte activity in inflammatory cells and polymorpho-nuclear phagocytes [4,5,6,7]. Oxidative stress, inflammation, and transporter abnormalities are thought to be the fundamental pathogenic pathways in the development of cholestasis and liver damage caused by biliary obstruction [8]. Some medications, including cyclosporine, may have cholestasis as a side effect [9]. It has been demonstrated that the immunosuppressant drug cyclosporine causes cholestasis by increasing the liver markers of hepato-cytolysis (ALT, AST) and the values of the cholestasis markers (ALP, direct bilirubin and γ-GT), in addition to increasing oxidative stress in rats. Calmodulin phosphatase inhibition is the primary mechanism by which cyclosporine achieves its specific activity. As a result, there is a buildup of calcium in the mitochondria, which leads to problems with the calcium pump and ATP homeostasis [10].

Antioxidants might help avoid liver damage brought on by obstructive cholestasis. It has been demonstrated in recent decades that a number of natural plants and their active constituents have protective and preventative benefits against cholestatic liver injury. These effects mostly involve reducing oxidative stress, fibrosis, inflammatory responses, and anomalies in bile acid metabolism. Natural products have been extensively researched and used because they are safer and have fewer side effects than traditional clinical hepato-protective medications. As a complementary therapy, they can offer guidance for the prevention and treatment of diseases [11]. By reducing oxidative stress and inflammation, natural products such as different flavonoids, phenols, acids, quinones, saponins, alkaloids, glycosides, gallic acid, and vitamin C have been shown to reduce cholestatic liver injury [12,13,14].

Pomegranate (*Punica granatum* L. Punicaceae family) is extensively grown in Mediterranean locations and has been used for medical intends since ancient times and throughout cultures. Numerous bioactive substances, including fatty acids, vitamin C and phenolic compounds, are found in pomegranates. Pomegranates’ phenolic components include flavonoids, phenolic acids, and hydrolyzable tannin. Gallic acid is the main phenolic acid found in pomegranates [15]. Traditionally, it has been used in Chinese medicine as an astringent and anti-inflammatory to treat bleeding, wounds, and gastrointestinal tract infections [16]. About 80% of pomegranate fruit is water, and 15% is made up of carbohydrates, primarily sugars like fructose, sucrose, and glucose. The remaining portion is made up of fiber, vitamins, and natural bioactive substances like polyphenols [17]. Pomegranate phytochemicals have anti-inflammatory, immunomodulatory and antioxidant activities [18]. According to Widyawat et al. [19], ellagic acid from whole pomegranate fruit improves liver enzyme profiles and reduces cholestasis that causes liver damage. Its antioxidant, anti-inflammatory, and anti-fibrotic properties may contribute to this.

Persimmon (*Diospyros kaki* L., Ebenaceae family) is highly recognized for its nutritional and therapeutic benefits, which are attributed to its abundance of minerals and phytochemicals. Persimmon, a nutrient- and antioxidant-rich fruit, contains a high concentration of proanthocyanidins, flavonoids (quercetin, catechins), phenolic acids (especially gallic acid), and vitamins (particularly vitamin C). It also contains carbs, carotenoids, and organic acids [20]. The proanthocyanidins found in persimmons, also called persimmon tannin, set them apart from other fruits [21]. According to Lalou et al. [22], traditional Chinese and Japanese medicine use fresh persimmon fruits or processed products (dried, juiced, and vinegared) to treat a variety of cardiovascular and immune system conditions. Numerous studies have demonstrated the anti-tumor, antioxidant, and anti-diabetic qualities of persimmon. Its abundance in antioxidants, such as vitamins, phenolic compounds, and carotenoids, has been linked to these health advantages [23].

Given that pomegranates and persimmons are fruits rich in the bioactive compounds phenolics, organic acids, and vitamin C, which provide strong antioxidant and anti-inflammatory activities, we hypothesized that they might play a role in combating cholestatic liver injury. To support this hypothesis, pomegranate and persimmon juices were separately evaluated for the protecting effect against oxidative stress and inflammation in cyclosporine-induced cholestasis in rats. This may support the use of these fruits as functional foods and may provide theoretical and application guidance for the hindrance of cholestatic liver damage.

## 2. Materials and Methods

### 2.1. Materials

Pomegranate and persimmon fruits were bought from a store that sold goods from the Egyptian Ministry of Agriculture in the Giza Governorate of Egypt. The capsules of cyclosporine, which are produced by Novartis Pharmaceutical Company under the trade name Neoral^®^, were purchased at a local pharmacy. There were 100 mg of cyclosporine in each capsule. The drug was dissolved in olive oil to create the required concentrations. We bought tripiridyltriazine, ascorbic acid, 2,2-diphenyl-1-picryl hydrazyl (DPPH), and ABTS (2,2′-azino-bis(3-ethylbenzothiazoline-6-sulfonic acid)) from Sigma-Aldrich Chemical Co. (St. Louis, MO, USA). This investigation used analytical grade chemicals and solvents.

### 2.2. Methods

#### 2.2.1. Pomegranate and Persimmon Juice Preparation

The fresh pomegranate and persimmon fruits were cleaned. A Black and Decker juice extractor (Baltimore, MD, USA) was used to compress the entire edible portion of the pomegranate (the seeds or arils and their connective sarcotestas). The persimmon fruits were sliced. Using an electric mixer (Panasonic, electric mixer grinder 1 L 1000 W Mx-ac210, Petaling Jaya, Malaysia), the persimmon pieces were pressed for approximately five minutes. The pomegranate and persimmon juices were separately filtered through cheesecloth, then the pH was measured using an analytical pH meter (pH 211, Hanna Instruments; made in Mumbai, India). Each juice was stored at −20 °C until utilization.

#### 2.2.2. Bioactive Compounds Determination

• Determination of vitamin C

To extract the sample, MilliQ water (10 mL) was added to each sample (2 g) in a 250 mL conical flask, then the conical flask was agitated for five minutes with a magnetic stirrer. After covering the flask with tin foil, it was sonicated for five minutes. For ten minutes, the solution was centrifuged (3000× *g*). The extracted samples were filtered through a 0.45 µm filter then injected into a high-performance liquid chromatography (HPLC) system.

Agilent 1260 series equipment (Agilent Technologies, Santa Clara, CA, USA) was used for HPLC analysis. The separation was conducted using Eclipse C8 column (Agilent, Santa Clara, CA, USA, 4.6 mm × 150 mm i.d., 5 μm). The mobile phase was 0.01%TFA: MeoH (70:30) at a flow rate 1 mL/min. Isocratic was the programming for the mobile phase. The multiple wavelength detector (MWD) detector was monitored at 248 nm. For every sample solution, 10 μL was the injection volume. The temperature in the column was set at 40 °C.

• Determination of total carotenoids content

Using spectrophotometry, the concentration of total carotenoids was determined using the Jamaleddine et al. [24] method. A total of 50 milliliters of ethanol was used to ultrasonicate 0.1 g of persimmon juice for one minute. Total carotenoids content was calculated by measuring the absorbance at 446 nm and was represented as milligrams of β-carotene equivalent per gram of fresh juice.

• Determination of organic acids

The sample (1 g) was magnetically stirred with 25 mL of an aqueous solution of HPO3 4.5% (*w*/*v*) for 20 min at 25 °C in order to extract and determine the organic acids. The sample was then filtered through a paper filter. Prior to chromatographic analysis, the extracts were once again filtered using a nylon syringe filter with a 0.2 µm pore size (Membrane Solutions, Kent, WA, USA). The HPLC 1260 series (Agilent Technologies, Santa Clara, CA, USA) was used to analyze organic acids. The SynergiTM 4 µm Hydro-RP 80 Å column (Phenomenex Inc., Torrance, CA, USA, 4.6 mm × 250 mm) was used to do the separation. A total of 20 mM potassium phosphate, pH 2.9, and a flow rate of 0.7 mL/min made up the mobile phase. At 220 nm, the Diode Array detector (DAD) was set. Each sample solution had an injection volume of 5 μL. The temperature of the column was kept at 22 °C.

• Determination of phenolic compound profile by HPLC

In brief, 0.5 g of each juice sample and 5 mL of methanol/water (70/30, *v*/*v*) were homogenized for 2 min in order to extract the sample. The mixture was then centrifuged for 10 min at 4 °C and 10,000 rpm. Following the separation of the supernatant, solid residues were extracted twice more using 3 mL of methanol/water (70/30, *v*/*v*) and once more using 3 mL of 100% methanol. After rotor-evaporating the obtained supernatants to a minimum volume at 25 °C (Buchi Labortechnik AG, Flawil, Switzerland), they were diluted with MilliQ water to a volume of 5 mL. For HPLC analysis, 1 mL of the extract was filtered through a 0.45 µm membrane. The rest of the extract’s volume was promptly frozen and kept at −20 °C until the antioxidant, total phenolic, and total flavonoid assays were completed.

An Agilent 1260 series HPLC was used to analyze phenolic chemicals. The separation was performed using the Zorbax Eclipse Plus C8 column (4.6 mm x× 250 mm i.d., 5 μm). The mobile phase consisted of water (A) and 0.05% trifluoroacetic acid in acetonitrile (B) at a flow rate of 0.9 mL/min. For the mobile phase, the ordered linear gradient programming periods were 0 min (82% A), 0–1 min (82% A), 1–11 min (75% A), 11–18 min (60% A), 18–22 min (82% A), and 22–24 min (82% A). The multi-wavelength detector was tuned at 280 nm. The injection volume of each sample solution was 5 μL. The column’s temperature was maintained at 40 °C. Gallic acid, chlorogenic acid, catechin, methyl gallate, coffeic acid, syringic acid, pyrocatechol, rutin, coumaric acid, vanillin, ferulic acid, naringenin, daidzein, querectin, cinnamic acid, apigenin, kaempferol, and hesperetin were the standard relative that was compared to the sample’s peak area to ascertain the concentration of each compound.

• Determination of the total phenolic content

Based on Singleton et al. [25], the phenolic concentrations of pomegranate and persimmon juice were determined using Folin–Ciocalteu and expressed as gallic acid equivalents (GAEs) per gram of sample. After adjusting the volume to 3.5 mL with distilled water, 50 μL of the extract was combined with 250 μL of Folin-Ciocalteau reagent. Five minutes later, the liquid was neutralized by adding 0.5 mL of a 20% aqueous sodium carbonate (NaCO_3_) solution. After 40 min, the absorbance at 765 nm was measured and contrasted with the solvent blank. Standard calibration curve of gallic acid (1.0, 2.5, 5.0, 10, 25, 50 and 100 µg/mL of gallic acid solutions) was used in the determination.

• Determination of the total flavonoid content

The total flavonoid concentration was ascertained by means of the aluminum chloride colorimetric assay, as stated by Campos et al. [26]. A total of 50 µL of extract was combined with 300 µL of sodium nitrite (5%). After 6 min of incubation, 300 μL of 10% aluminum chloride solution was added, and distilled water was added to reach 1.80 mL. After that, 1.5 mL of 1 M NaOH was added to the mixture. At 420 nm, the supernatant’s absorbance was measured in relation to the solvent blank. Using a calibration curve created using catechin, the total flavonoid concentration was calculated and reported as milligrams of catechin equivalent (mg CE) per gram sample.

• Determination of antioxidant activity

Three techniques were used to evaluate the ability of the extracts from pomegranate and persimmon juices to scavenge free radicals: DPPH radical scavenging assay, ABTS radical cation assay, and FRAP (ferric ion reducing antioxidant power).

The assay for DPPH radical scavenging was conducted in compliance with Brand-Williams et al. [27]. After adding one milliliter of each extract to the DPPH solution, the mixture was left to incubate for half an hour. A spectrophotometer was then used to detect the solution’s absorbance at 517 nm. Three different assays were conducted. The following formula was used to determine the percentage of DPPH radical scavenging activity:

(1) DPPH radical scavenging activity (%) = (A_c_ − A_s_/A_c_) × 100

where A_s_ is the absorbance of the sample and A_c_ is the absorbance of the control in the absence of the sample.

When conducting the ABTS radical cation assay, the protocol described by Prior et al. [28] was adhered to. Prior to use, the ABTS+ cation radical was produced by mixing 10 mg of ABTS with 2 mg of potassium persulfate in water and letting it sit in the dark for 12–16 h. The absorbance at 734 nm was measured thirty minutes after 5 μL of each extract was added to 3.995 mL of diluted ABTS+ solution. The ABTS+ solution (1 mL) was then diluted with 60 mL of methanol. Three different assays were conducted. The following formula was used to calculate the percentage of ABTS scavenging activity:

(2) ABTS scavenging (%) = (A_c_ − A_s_/A_c_) × 100

where A_s_ is the absorbance of the sample and A_c_ is the absorbance of the control in the absence of the sample.

The FRAP assay was carried out according to Gomez et al. [29]. The FRAP reagent was made by combining 300 mM acetate buffer, 10 mL tripiridyltriazine in 40 mM hydrochloric acid, and 20 mM ferric chloride (FeCl_3_6H_2_O) in a 10:1:1 ratio at 37 °C. Five µL of each extract were blended well with 3.995 mL of FRAP reagent. A bright blue complex was created in the reaction mixture when ferric tripiridyltriazine was reduced to a ferrous tripiridyltriazine form. The absorbance at 593 nm was detected after 30 min of keeping at 37 °C using a reagent blank consisting of 5 μL of distilled water and 3.995 mL of FRAP reagent. Three separate assays were run. A calibration curve with different FeSO_4_ concentrations could be made by plotting the absorbance at 593 nm.

#### 2.2.3. Bioactive Potential Against Cholestatic Liver Injury

• Animals

Male Wistar albino rats (*Rattus norvegicus*) that were healthy and mature (6 weeks) weighed 157.55 ± 6.95 g (mean ± SD) and were acquired from the animal house of the National Research Center in Cairo. A total of 12 h light/dark cycle, regulated humidity (50 ± 10%), and temperature (22 ± 2 °C) were used to house rats. During the trial, all rats had unrestricted access to food and water. According to Reeves et al. [30], a balanced diet including 12% casein, 10% corn oil, 10% sucrose, 58.5% maize starch, 5% fiber, 3.5% AIN-93 salt mixture, and 1% AIN-93 vitamin mixture was created. The study methodology was authorized by the Medical Research Ethics Committee (MREC) of the National Research Center in Cairo, Egypt, which also made sure that every animal was treated humanely (Code 14921122022).

• Grouping and treatments

In accordance with the schedule and after being acclimated to laboratory conditions for a week prior to the start, twenty-four rats were randomly assigned to four groups (n = 6) and given various treatments:

1. The control normal group (N) was given 1 mL of saline (vehicle) orally for 4 weeks.
2. The cyclosporine group (Cs, positive control) was given a daily dose of 15 mg/kg body weight of cyclosporine dissolved in 1 mL of olive oil by gavage for 4 weeks [31,32].
3. The third group (P) was given a daily dose of 15 mg/kg body weight of cyclosporine dissolved in 1 mL of olive oil along with pomegranate juice (the dose that provides 50 mg of gallic acid equivalent/kg/day) by gavage for 4 weeks.
4. The fourth group (D) was given a daily dose of 15 mg/kg body weight of cyclosporine dissolved in 1 mL of olive oil along with persimmon juice (the dose that provides 50 mg of gallic acid equivalent/kg/day) by gavage for 4 weeks.

Given the different concentrations of bioactive compounds in both pomegranate and persimmon juices, and on the basis that phenolics are the major bioactive compounds in both juices that provide strong antioxidant activity, the dosage of the two juices was determined based on the quantity that provides the same amount of total phenolics (equivalent to gallic acid). The dosage of pomegranate and persimmon juices was chosen based on Attia et al. [33]. Throughout the experiment duration (4 weeks) and during the daily oral administration, food consumption was determined every day, and body weight and overall health status were monitored. The calculations of total food intake, body weight gain, and feed efficiency ratio were carried out at the end of the experiment. None of the rats died during the experiment. Blood samples were obtained from rats that had been sufficiently anesthetized using sodium pentobarbital (50 mg/kg, i.p.). The blood was collected by retro-orbital sinus bleeding, then animals were euthanized by cervical dislocation under deep anesthesia and the livers were removed through dissection. Serum was taken away from the blood samples, through the centrifuging for 15 min at 3500 rpm, then kept at −80°C until analysis. For the histological analysis, a part of the liver was submerged in formalin–saline (10%). In a cold potassium phosphate buffer (100 mM, pH 7.4), a different part of the liver was homogenized (10% *w*/*v*). After centrifuging the homogenized samples, the supernatants were gathered and utilized for biochemical examination.

• Biochemical analysis

Using the colorimetric techniques, the plasma of each rat was assayed for total protein and the activities of gamma-GT (γ-GT), alkaline phosphatase (ALP), lactate dehydrogenase (LDH), and aspartate transaminase (AST) [34,35,36,37,38]. The levels of total and direct bilirubin were determined depending on [39]. According to the manufacturer’s instructions, Elisa kits (Sunlong Co., Ltd., Shanghai, China) were used to estimate liver tumor necrosis factor-alpha (TNF-α), interleukin-6 (IL-6), interleukin 1-beta (IL-1β), and toll-like receptor 4 (TLR4), as well as plasma immunoglobulin G (IgG) and immunoglobulin M (IgM). Using the colorimetric techniques outlined by [40,41,42,43], the levels of liver superoxide dismutase (SOD) activity, nitric oxide (NO), reduced glutathione (GSH), and malondialdehyde (MDA) were measured.

• Gas chromatography–mass spectrometry (GC-MS) analysis of serum metabolites

Serum sample metabolites were separated and derivatized using the methodology of Shouk et al. [44]. In summary, the proteins were precipitated by centrifuging 200 μL of acetonitrile, 100 μL of serum, and 5 μL of xylitol (1 mg/mL) as an internal standard for 15 min at 11,000× *g*. Following the collection and freeze-drying of the supernatant, the dried metabolite pellets were mixed with 50 μL of methoxyamine HCl (20 mg/mL in pyridine) and heated to 60 °C for two hours. Next, 100 μL of MSTFA with 1% TMS was added, and the mixture was kept at 60 °C for an hour.

Serum metabolites were profiled using a Shimadzu GC-MS-QP2010 (Kyoto, Japan) in conjunction with a Single Quadrupole Mass Spectrometer detector. Derivatized serum metabolites were separated on a Rtx-5MS fused bonded column (0.25 μm film thickness × 0.25 mm i.d. × 30 m) (Restek, Bellefonte, PA, USA) using helium as the carrier gas at a flow rate of 1 mL/min. The temperature was raised to 290 °C at a rate of 5 °C per minute after two minutes at the initial column temperature of 80 °C. This temperature was then sustained for five minutes. The injector and detector had a fixed temperature of 280 °C. The injection volume was 1 μL with a spectral range of *m*/*z* 40–600, and the mass spectra were acquired using electron ionization (EI) at 70 eV.

• Processing of GC–MS data and multivariate data analyses

MZMine v.2.9.1, a free program, was used to process GC-MS raw data. These data included peak filtering operations like alignment, retention time, mass-to-charge ratio, and peak area. The Human Metabolome Database (https://hmdb.ca/, accessed on 1 January 2026), Golm Metabolome (http://gmd.mpimp-golm.mpg.de/, accessed on 1 January 2026), and the NIST Library 2005 “National Institute of Standards and Technology” were among the databases used to identify serum metabolites. Following Excel normalization, the datasets were loaded into SIMCA 14.1 (Umea, Sweden) software, where multivariate statistical analyses were conducted using principal component analysis (PCA) and orthogonal partial least-squares discriminant analysis (OPLS-DA).

• Histopathological examination

Each liver’s hepatic lobules underwent three steps: immersion in 4% paraformaldehyde, gradient alcohol dehydration, paraffin embedding, slicing, dewaxing, and rehydrating. The Olympus IX83 microscope (Tokyo, Japan) was used to view each segment after it had been stained with hematoxylin–eosin staining (H&E).

• Evaluation of the anti-inflammatory effects through molecular docking assay

Using the molecular docking assay, the anti-inflammatory properties of persimmon and pomegranate juices were investigated. Using the PyRx tool (version 3.12.2) [45,46], the binding affinity score of gallic acid, one of the primary bioactive compounds found in pomegranate and persimmon juices, on the active pocket of the inflammatory cytokines human toll-like receptor 4 (TLR4) (PDB: 3UL7) and human tumor necrosis factor (TNF-alpha) (PDB: 7JRA) was ascertained. The PubChem database “https://pubchem.ncbi.nlm.nih.gov/” (accessed on 17 January 2026) provided the 3D chemical structures of gallic acid and catechin (SMILE code), which were then created using the Chem Draw (version 12.0) program. After being loaded into the PyRx program, the aimed proteins and ligands were transformed into PDBQT [45]. The docking assay was conducted after grid boxes with dimensions of 25 × 25 × 25 Å were placed at the co-crystallized ligands to cover all of the target proteins’ binding sites [46]. The protein–ligand complex was visualized using PyMOl (version 2.5.5) and Discovery Studio (version 2021) (3D and 2D).

#### 2.2.4. Statistical Analysis

SPSS version 21 (SPSS Inc., Chicago, IL, USA) was used for statistical analysis. The results of all bioactive compounds of both juices were expressed as mean ± standard deviation (SD), and the data were statistical analyzed using an independent samples *t*-test. One-way analysis of variance (ANOVA) was used to statistically examine the data of the in vivo assay, and the findings were presented as mean ± standard error of means (SEM). The statistical differences between the groups were examined using the Duncan test. *p* < 0.05 was used to establish the difference’s statistical significance.

## 3. Results and Discussion

### 3.1. Bioactive Compounds in Pomegranate and Persimmon Juices

The yield from pomegranate juice was 32%, while the yield from persimmon juice was 46%. The pH of the pomegranate juice was 3.2, and that of the persimmon juice was 5.7.

#### 3.1.1. Total Caroteniod Content

Compared to persimmon juice, pomegranate juice contained a lower concentration of carotenoids (2.73 ± 0.04 as mean ± SD µg β-carotene per gram), while persimmon juice had a total carotenoids level of 0.16 ± 0.015 (mean ± SD) mg β-carotene per gram on a fresh weight basis. Lipophilic pigments known as carotenoids are found naturally in plants and, to a smaller degree, in some non-photosynthetic species. Because of their immunomodulatory, anti-inflammatory and antioxidant activities, they are vital to human health. According to evidence, these substances may lessen pro-inflammatory effects and cytokine storms, primarily via the JAK/STAT and NF-κB signaling pathways [47]. Nutrients and bioactive compounds abound in persimmon fruit. They are popular with consumers because of their antioxidant capacity, which are mainly ascribed to flavonoids, phenolic compounds, and carotenoids [48]. In comparison to other studies, such as the one conducted by Ann et al. [49], which found that there was 0.0025 mg of β-carotene per gram of persimmon fruit, the level of total carotenoids in our results is a high amount. According to El Moujahed et al.’s [50] study, the total carotenoids in the juice of different varieties of pomegranate ranged from 1.2 to 18.7 µg/mL (beta-carotene equivalents).

#### 3.1.2. Vitamin C

**Figure 1:**
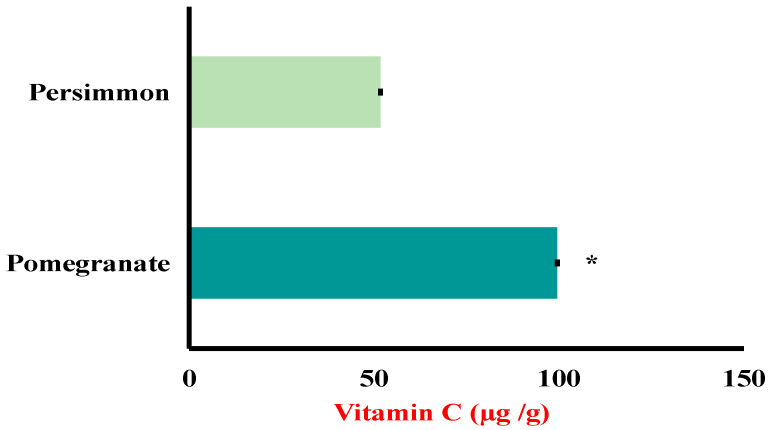
Pomegranate and persimmon contents of vitamin C. The symbol * refers to the significant difference using *t*-test.

**Figure 2:**
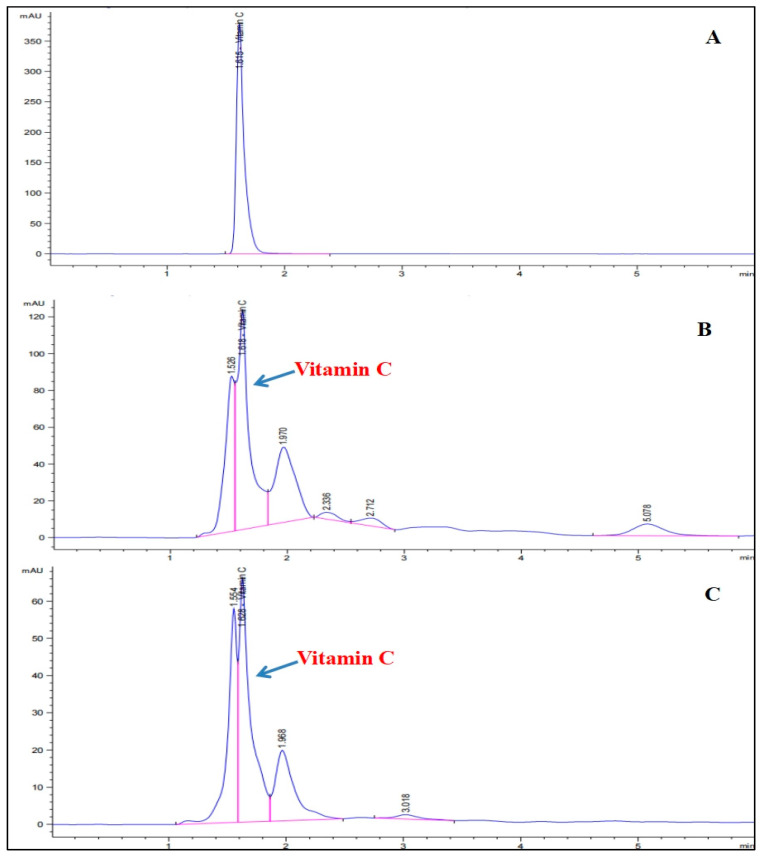
HPLC chromatograms of vitamin C levels. (**A**) The used standard, (**B**) pomegranate juice, (**C**) persimmon juice.

Pomegranate and persimmon juices had vitamin C contents of 99.55 ± 0.04 and 51.75 ± 0.03 µg per gram (mean ± SD), respectively, as demonstrated quantitatively in Figure 1 and illustratively in the chromatograms (Figure 2B,C). The chromatogram (Figure 2A) is for vitamin C used as a standard, injected (50 µg/mL) into the HPLC. Because they are rich in water-soluble vitamins like vitamin C, pomegranates and persimmons are recognized to have a higher antioxidant capacity than other fruits. Water-soluble vitamin C is a potent antioxidant [51]. A credible hepato-protective effect was shown by vitamin C [52]. It prevents microsomal lipid peroxidation, liver fibrosis, liver necrosis, and hepatic inflammation by increasing the activities of superoxide dismutase, catalase glutathione peroxidase, and free radical scavengers [53].

#### 3.1.3. Organic Acid Composition

**Figure 3:**
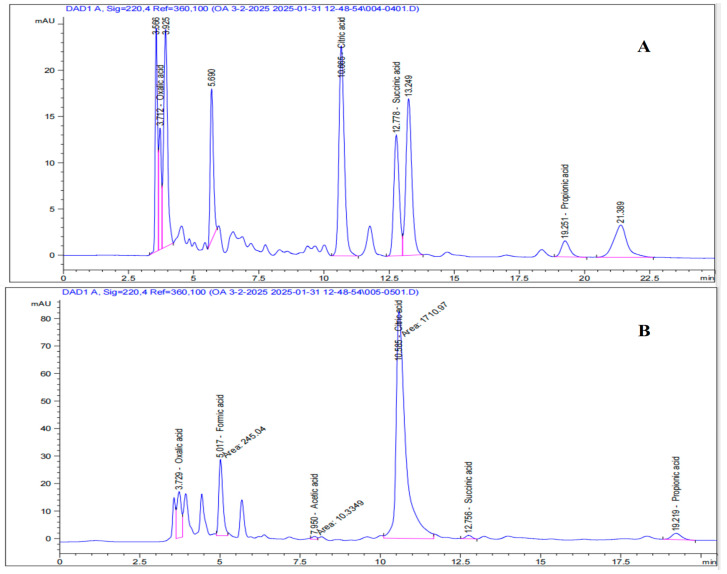
HPLC chromatograms of organic acid contents: (**A**) pomegranate juice, (**B**) persimmon juice.

The findings of characterizing the organic acid composition of the pomegranate and persimmon juices are displayed quantitatively in Table 1 and illustratively in the chromatograms (Figure 3). Citric acid was the most prevalent of the six organic acids found in pomegranate juice (10,272.24 µg/g fresh weight), followed by formic acid (1781.78 µg/g), then propionic acid (642.15 µg/g). A lower quantity of oxalic acid (118.52 µg/g) was measured. According to results, the primary organic acid in persimmon fruit was succinic acid (2699.24 µg/g fresh weight), which was followed by citric (1946.67 µg/g) and propionic acids (508.63 µg/g). Conversely, lower concentrations of oxalic acid (67.33 µg/g) have been reported. In the same line of our findings, Nafees et al. [54] explored that pomegranate juice contained a variety of organic acids, such as malic, citric, succinic, oxalic, and tartaric acids. Additionally, another study reported that the sour flavor of pomegranate juice is intimately linked to both citric and malic acids. According to [55], malic acid and citric acid were established to be the main organic acids in persimmon fruit, with lower quantities of succinic, tartaric, and oxalic acid. Lipid peroxidation is prevented by the antioxidant activity of certain organic acids. Additionally, it has been discovered that organic acids, particularly citric acid, may work in concert with non-phenolic substances as molecules that scavenge DPPH radical. According to earlier research, some organic acids directly affect how well plant extracts scavenge free radicals [56].

#### 3.1.4. Phenolic Compounds Profile

**Figure 4:**
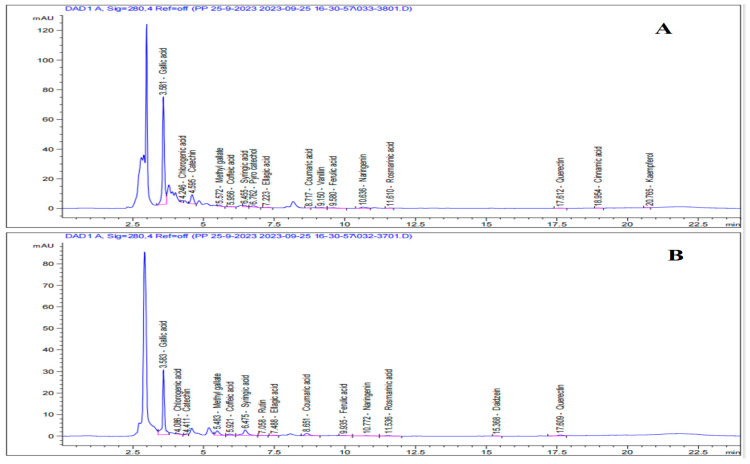
HPLC chromatograms pf phenolic profile. (**A**) Pomegranate juice, (**B**) persimmon juice.

The findings (Table 2 and Figure 4) point to the fact that gallic acid (123.20 µg/g fresh weight) and catechin (37.78 µg/g) were the two most prevalent phenolic compounds found in pomegranate juice. Similarly, gallic acid (50.69 µg/g fresh weight) and syringic acid (9.68 µg/g) have been identified as the main phenolic constituents in persimmon fruit. According to Shahkoomahally et al. [57], gallic acid and ellagic acid are two important phenolic acids that are present in significant proportions in pomegranate juice. The phenolic profile of various persimmon types revealed that gallic acid and catechin were the most significant components [58]. Gallic acid can activate nuclear erythroid 2-related factor 2, a crucial transcription factor that regulates the expression of antioxidant enzymes. Gallic acid can also alter certain signaling pathways associated with oxidative stress and inflammation, such as nuclear factor kappa B pathways, by triggering the Keap1/Nrf2/ARE pathway, which leads to the production of proteins that block the IKK complex. This inhibition reduces the inflammatory response and stops NF-κB activation [59].

#### 3.1.5. Total Phenolic and Flavonoid Contents as Well as Antioxidant Activity

**Figure 5:**
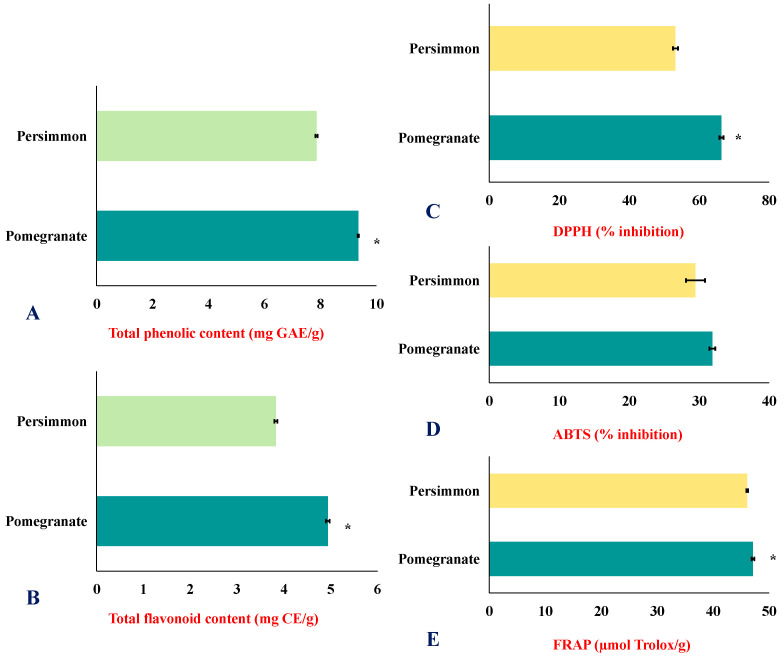
Pomegranate and persimmon juices’ TPC, TFC, and antioxidant properties (mean ± SD). TPC (**A**), TFC (**B**), DPPH inhibition % (**C**), ABTS inhibition % (**D**), and FRAP (**E**). The symbol * refers to the significant difference using *t*-test.

Figure 5A–E display the total phenolic content, total flavonoids content, DPPH scavenging activity, ABTS scavenging activity and FRAP of pomegranate and persimmon juices. Pomegranate juice had the highest total phenolic content (9.35 ± 0.02 mg GAE/g) and the highest flavonoid content (4.94 ± 0.04 mg CE/g), as well as the highest antioxidant activity (DPPH; 66.37 ± 0.58%, ABTS; 31.87 ± 0.42%, FRAP; 47.11 ± 0.24 µmol Trolox/g). Persimmon fruit had antioxidant activity (DPPH: 53.22 ± 0.71%, ABTS: 26.46 ± 1.36%, FRAP: 46.06 ± 0.15 µmol Trolox/g), as well as total phenolic content equal to 7.86 ± 0.04 mg GAE/g and flavonoid content equal to 3.83 ± 0.03 mg CE/g. The antioxidant effects of pomegranates can be attributed to their phenolic components, which include polyphenols, flavonoids, flavanols, and anthocyanins [60]. Cairone et al. [61] found that packaged pomegranate juice using pasteurization and high-pressure processing recorded TPC equal to 15.9 ± 0.16 and 11.5 ± 1.53 mg GAE/g, TFC equal to 5.0 ± 0.20 and 1.9 ± 0.48 mg RE/g, DPPH equal to 55.1 ± 0.74 and 35.1 ± 0.74 mg TE/g, ABTS equal to 91.8 ± 0.78 and 51.5 ± 1.71 mg TE/g, and FRAP equal to 75.3 ± 1.04 and 48.2 ± 1.13 mg TE, respectively. Persimmon has a higher antioxidant capacity than other fruits because it contains more carotenoids, phenolic compounds (especially tannin), and water-soluble vitamins such as vitamin C. Several studies have shown that persimmons have a considerably higher antioxidant capacity than other fruits, such as apples [62]. The extract of persimmons from fresh fruits from the Hadong region indicated antioxidant activity, as it included considerable quantities of total phenolic (298.01 mg GAE/kg) and flavonoid (32.11 mg/kg RE) constituents.

### 3.2. Findings of the In Vivo Assay

#### 3.2.1. Effect of Pomegranate and Persimmon Juices on Liver Injury Caused by Cyclosporine

In this study, and following several previous studies, cyclosporine A was chosen to induce liver damage and cholestasis in a rat model. Cyclosporine is an immunosuppressant drug after organ transplantation in humans, but cholestatic liver damage led to limiting its use as a drug in preventing organ rejection after organ transplantation [63,64]. According to Ommati et al. [65], hepatic injury caused by cholestasis is largely caused by oxidative stress and mitochondrial dysfunction. Therefore, cholestasis-induced damage may be mitigated by administering compounds with antioxidant qualities and the ability to shield mitochondria. Compared to medication therapy, natural plants have a better safety record for protection from the oxidative stress mediated by cyclosporine. Additionally, it has been demonstrated that the active constituents of plants have hepato-protective properties, which makes them suitable supplements for the treatment or prevention of clinical conditions such cholestasis and subsequent damage [66]. Pomegranate and persimmon are abundant in antioxidants, including vitamins, flavonoids, and polyphenols, which have strong anti-inflammatory properties, combat oxidative stress, and may offer protection against the side effect of cyclosporine and cholestatic liver injury. In this study, the juices of these fruits were chosen as they are easily prepared, and easily consumed in fresh or packaged form.

• Effect on body weight and food intake

Following a month of cyclosporine treatment, the Cs group’s average weight gain and total food intake were significantly (*p* < 0.05) lower than those of the N group, as indicated by the results (Table 3). This suggested that cyclosporine had a detrimental effect on the liver, which was evident in growth and body weight. Group Cs exhibited a considerably (*p* < 0.05) higher liver weight than the other groups, although they recorded a lower significant weight than the N group. Rats given pomegranate juice gained more weight and had significantly less liver weight than the Cs group. Rats which had taken persimmons had significantly (*p* < 0.05) less liver weight than the Cs group, but they did not significantly vary (*p* > 0.05) in their overall weight gain from the Cs group. However, because persimmon fruit is high in fiber and pectin, it has the ability to reduce body weight [21].

• Effect on the liver functions and indicators

**Figure 6:**
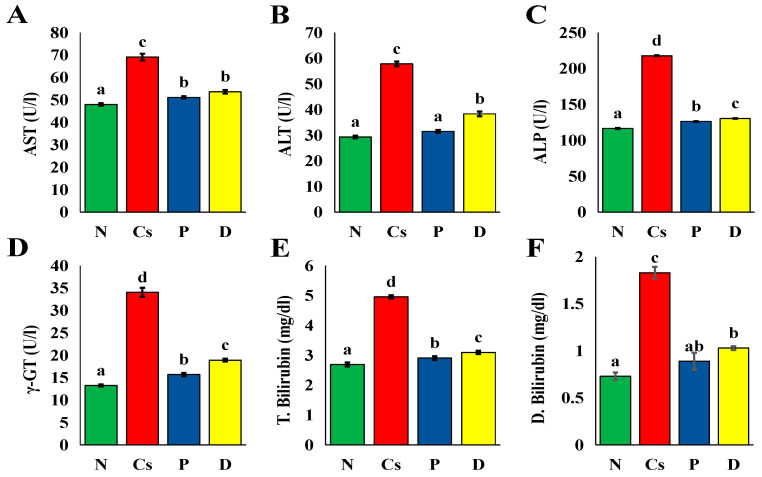
The liver functions and indicators. (**A**) AST activity, (**B**) ALT activity, (**C**) ALP activity, (**D**) γ-GT activity, (**E**) total, (**F**) direct bilirubin level. The mean ± SEM (n = 6) is used to express all data. There is a substantial difference (*p* < 0.05) between mean values with distinct letters (a ˂ ab ˂ b ˂ c ˂ d). N stands for normal; Cs for cyclosporine; P for pomegranate; and D for persimmon.

Figure 6 displays the values of the markers and liver functions. In the same line with Qin et al. [64], the values of the liver markers of hepato-cytolysis (ALT, AST) and the values of the cholestasis markers (ALP, direct bilirubin and γ-GT) were considerably higher after the administration of cyclosporine in the Cs group (*p* < 0.05) than in the normal rats, but they were the opposite in the groups that received pomegranate and persimmon juices (*p* < 0.05). Pomegranate juice outperformed persimmon juice in its ability to lower AST, ALP, and γ-GT levels. The Cs group had considerably higher levels of both total and direct bilirubin (*p* < 0.05) than group N. The elevation in ALP, direct bilirubin and γ-GT levels after the administration of cyclosporine may indicate the cholestasis induction. According to [67], only an increase in serum ALP and γ-GT levels are signs of the early stage of cholestasis in humans. Elevated direct bilirubin, the cause of jaundice and dark urine, is a biochemical indicator of cholestasis, or impeded bile flow [68]. In contrast, the elevations in AST, ALT, ALP, direct bilirubin and γ-GT levels were inhibited by pomegranate and persimmon juice intervention. This may indicate the hepato-protective effect of both juices. However, pomegranate juice led to a reduction in direct bilirubin levels that was not considerably (*p* < 0.05) different from the N group.

Antioxidant depletion and reduced liver function are linked to mitochondrial damage and elevated reactive oxygen species (ROS), which causes cell impairment. Thus, because of the substantial ROS buildup brought on by cyclosporine toxicity, mitochondrial damage is indirectly correlated with a rise in liver cell injury markers such as ALT, AST, and bilirubin [10]. The human liver contains a large amount of the enzyme ALP, which the liver then releases into the bile, as confirmed by Sun et al. [11]. It functions as an unambiguous marker for determining the extent of biliary damage and the pathological process of cholestasis. When bile cannot be expelled regularly, a great deal of ALP is generated. Rats treated with cyclosporine showed a marked increase in ALP activity, a sign of obstructed bile excretion. Pomegranate and persimmon juices reversed this effect, indicating that pomegranate and persimmon may help cure cholestatic liver damage caused by cyclosporine. Pomegranate and persimmon juices may have an inhibitory effect on liver damage. This may be attributed to the content of vitamin C since Qutb et al. [53] discovered that vitamin C-loaded hydrogel reduced DNA damage in cholestatic rats, partly fixed the aberrant hepatic tissue construction, and reduced inflammation by lowering interleukin 6 and interleukin 1β concentrations. Additionally, Soylu et al. [14] reported that vitamin C intervention decreased ALP, directed bilirubin and γ-GT levels and modified hepatic fibrosis in biliary-obstructed rats. Gallic acid may also contribute to the hepato-protective effect of pomegranate and persimmon juice; Jafaripour et al. [13] explored that gallic acid ameliorated liver cirrhosis by a reduction in oxidative stress and fibrogenesis in the liver of biliary-obstructed rats. Not only vitamin C and gallic acid but also other antioxidants in pomegranate and persimmon juices, including organic acids and phenolics, may be involved in reducing cyclosporine-induced hepato-toxicity and cholestatic liver injury [10]. According to Zamanian et al. [69], pomegranate polyphenols can preserve liver enzymes by raising paraoxonase 1 mRNA and protein levels in the liver. Consuming pomegranate fruit, which is rich in antioxidants, can boost overall antioxidant capacity, lower TNF-α, a measure of inflammation, and reduce the activity of AST and ALT, markers of liver damage. According to research by Kuang et al. [70], persimmon juice not only has a strong antioxidant capacity but also offers more thorough liver protection by controlling lipid metabolic homeostasis and inhibiting ferroptosis.

• Serum metabolites profile

**Figure 7:**
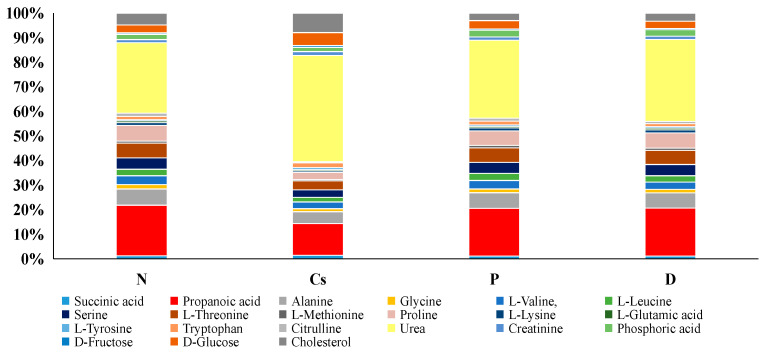
Percentage of some metabolites detected by GC-MS for blood of the studied groups. (n = 3). N stands for normal; Cs for cyclosporine; P for pomegranate; and D for persimmon.

Following cyclosporine treatment, amino acid metabolisms were dramatically affected, according to the thorough serum metabolites study (Figure 7 and Table 4). Most amino acids were significantly less in the Cs group. In contrast, the pomegranate and persimmon-treated groups recorded higher levels of most of the amino acids. The other serum metabolites (cholesterol, glucose, fructose, urea and creatinine) were significantly higher in the Cs group. However, compared to the Cs group, the pomegranate and persimmon-treated groups had considerably lower levels of cholesterol, glucose, fructose, urea and creatinine. The findings of the serum samples may also indicate the hepato-protective impact of pomegranate and persimmon juices and the detrimental effect of cyclosporine.

The liver contains the majority of the enzymes involved in the metabolism of amino acids. Protein synthesis may be disrupted by damage to the liver tissue; the amount of amino acids may drop when they are oxidized due to a defect in protein production, which will affect the liver’s regular physiological function [66]. Leucine, isoleucine, and valine are branched-chain amino acids that are depleted in liver injury. Supplementing with these amino acids has been suggested as a way to reduce liver fibrosis and enhance regeneration [71]. Along with problems with amino acid metabolism, urea levels rose in the Cs group, indicating impaired liver and kidney function. The primary byproduct of amino acid catabolism is urea, and high blood urea concentration can be a sign of compromised renal function [72]. Conversely, rats that received pomegranate and persimmon juices demonstrated a rise in amino acids and a fall in urea, suggesting that these fruits have a protective impact on the liver and kidneys. It was discovered that the Cs group had higher levels of creatinine, a metabolite associated with energy metabolism, than the N group. The non-enzymatic breakdown of phosphocreatine produces creatinine, and the creatinine–phosphocreatine pathway is recognized to be essential for the movement of energy inside cells [73]. Furthermore, creatinine is frequently used as a common indicator of renal dysfunction [74]. Significant hypercholesterolemia is frequently brought on by cholestasis, which manifests as high serum total cholesterol and, more especially, aberrant low-density lipoprotein. This disorder results in an abnormal lipid profile because cholesterol re-fluxes from the liver into the bloodstream instead of being eliminated [75].

**Figure 8:**
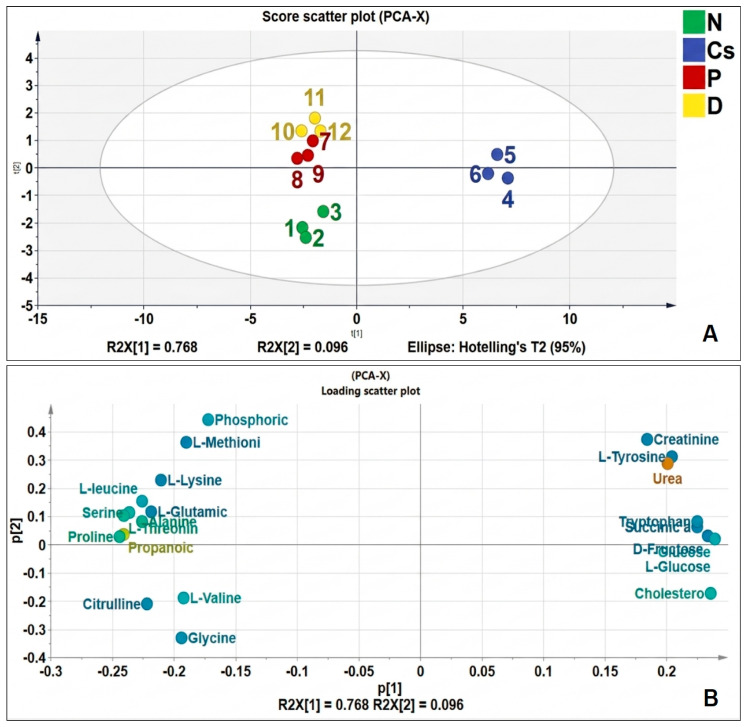
PCA of serum metabolites. (**A**) score scatter plot, (**B**) loading. (n = 3). N stands for normal; Cs for cyclosporine; P for pomegranate; and D for persimmon.

This study employed the principle component analysis (PCA) multivariate analysis approach to show how the groups under the study differed and were similar. The scatter plot of PCA scores is shown in Figure 8A. As we can see, a sufficient separation was demonstrated among the four groups, which indicated that pomegranate and persimmon juices significantly altered the levels of endogenous metabolites in rat serum. Figure 8B is the loading of PCA, as shown in a loading diagram, also known as a correlation diagram. The coordinates of each variable correspond to the correlation and directive with PC1 and PC2, respectively. R2X (1) and R2X (2) are the explanatory rates corresponding to principal components 1 and 2. It can be seen from Figure 8 that R 2X (1) = 0.768 and R2X (2) = 0.096, indicating that the contribution of the two principal components are great.

**Figure 9:**
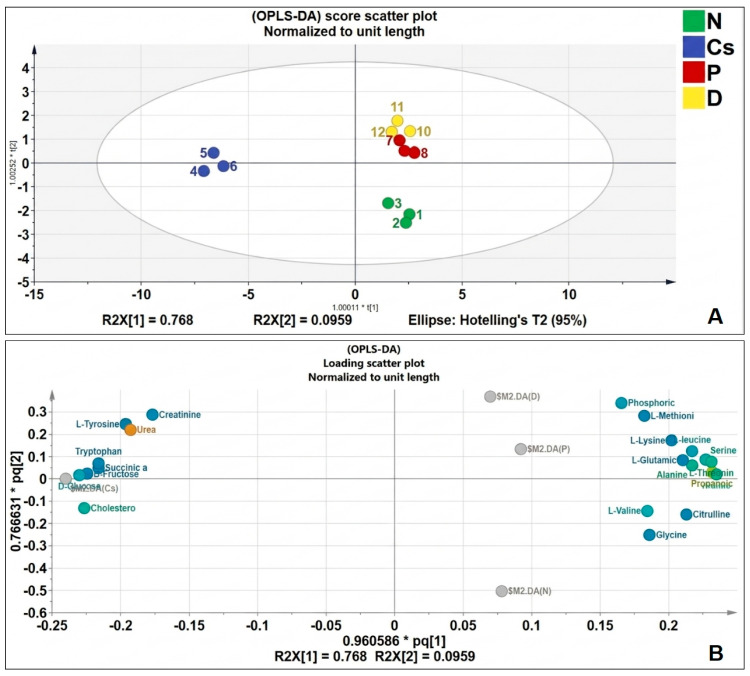
OPLS-DA of serum metabolites. (**A**) Score scatter plot. (**B**) Loading. (n = 3). N stands for normal; Cs for cyclosporine; P for pomegranate; and D for persimmon.

To further investigate whether pomegranate and persimmon juices may protect the liver from damage mediated by cyclosporine, the groups under investigation were subjected to an orthogonal partial least-squares discriminant analysis (OPLS-DA) model (Figure 9A,B). Differences between the Cs group and the normal group were displayed in the score scatter plot (Figure 9A). The persimmon and pomegranate groups were closer to the N group and more densely packed together. Figure 9B illustrates the loading of the score scatter plot normalized to the unit length.

**Figure 10:**
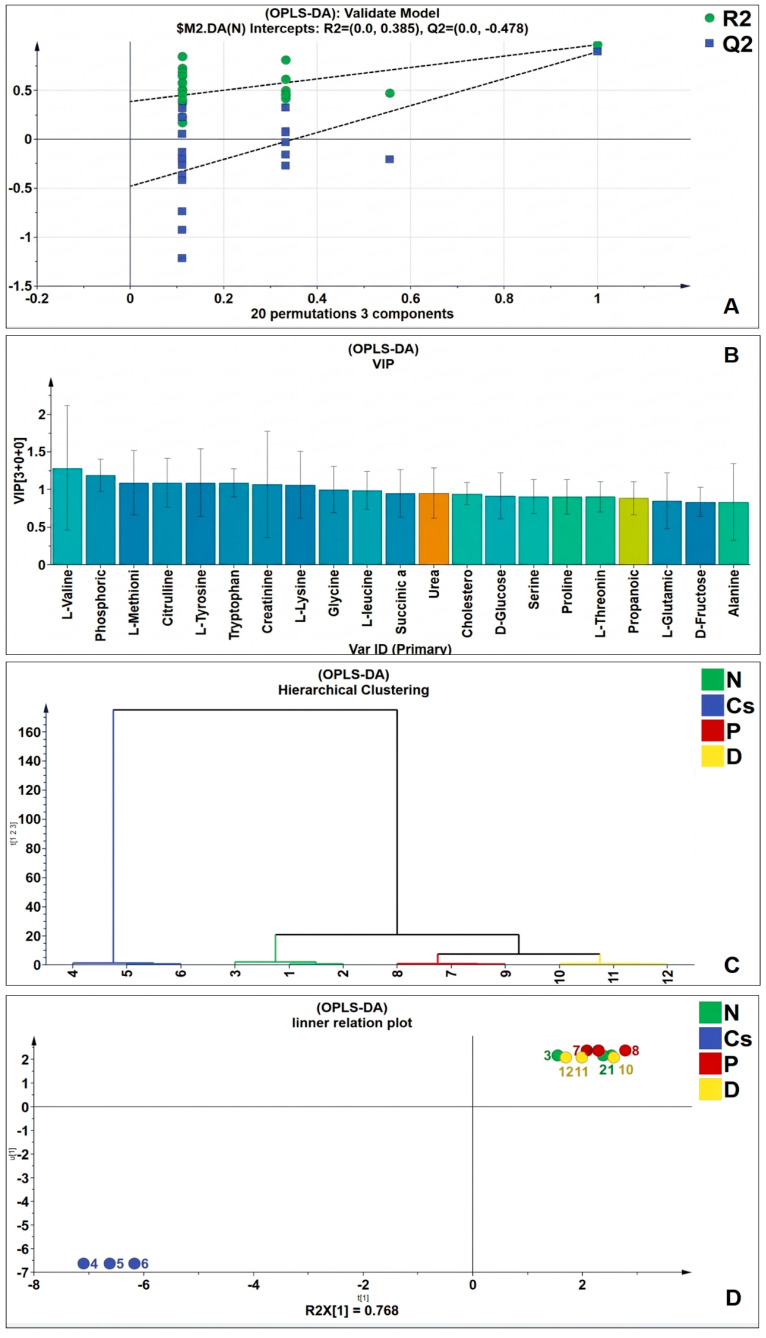
OPLS-DA of serum metabolites. (**A**) random replacement test, (**B**) VIP scores, (**C**) the inner relationship between the studied groups, (**D**) hierarchical cluster analysis (HCA). (n = 3). N stands for normal; Cs for cyclosporine; P for pomegranate; and D for persimmon.

Under the recognized OPLS-DA model, the random replacement test, the explanatory rate (R2y), prediction ability (Q2) and other parameters were assessed, the OPLS-DA model displayed acceptable cross-validation parameters, being R2X (cum) = 0.9, R2Y (cum) = 0.914 and Q2Y = 0.707 and the results of the permutation test cross-validation were R2 = 0.385, Q2 = −0.478 (Figure 10A). The results demonstrate that the model had strong stability and prediction capacity and there was no over fitting.

Valine, phosphoric acid, methionine, citrulline, tyrosine, tryptophan, creatinine, and lysine were the most discriminant metabolites, with a VIP score > 1, according to the OPLS-DA model’s variable importance in projection (VIP) (Figure 10B). There is a clear division between the four groups, according to the data of the hierarchical clustering test (Figure 10C) and the inner relation plot (Figure 10D). The persimmon and pomegranate groups were far from the Cs group and closer to the N group and more densely packed together. As a result, rats given cyclosporine had considerably different quantities of endogenic metabolites in their serum.

• Histopathological findings.

**Figure 11:**
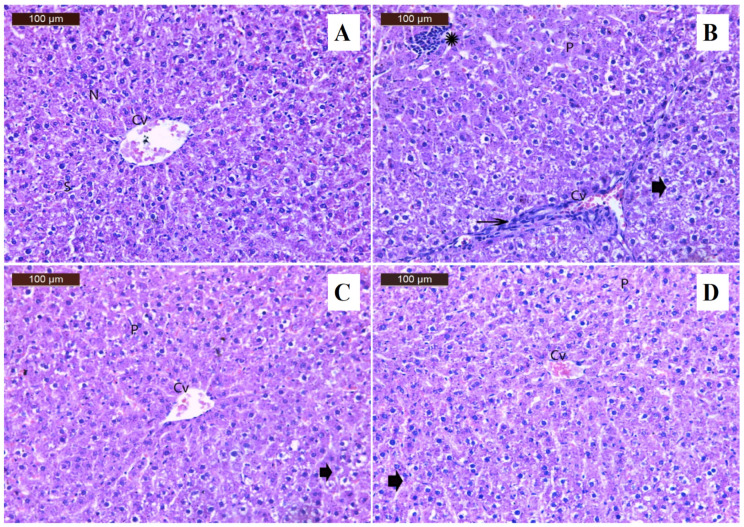
The photomicrographs of the liver tissues (stained by H&E, 200×) from the studied groups. (**A**) Normal group with normal structure, (**B**) cyclosporine group with degeneration in hepatocytes of liver (arrowhead), dilatation of the central vein with inflammatory cell infiltration (arrow), focal inflammatory cell (star) and pyknotic nuclei, (**C**) pomegranate group with moderate degeneration in hepatocytes of liver (arrowhead), (**D**) persimmon group with mild degeneration in hepatocytes of liver (arrowhead). CV = central vein; S = sinusoids; nucleus (N); P = pyknotic nuclei.

A histological image of the liver (Figure 11A) of the N group revealed that the liver tissues, including the nucleus, hepatic sinusoids, and central vein, were normal in structure. The cyclosporine treatment caused changes in the liver tissues compared to the N group, including focal inflammatory cell and pyknotic nuclei, dilatation of the central vein in addition to inflammatory cell infiltration, and degradation in the liver’s hepatocytes (Figure 11B). The liver sections of the group that had cyclosporine and pomegranate treatment displayed a normal central vein, a few pyknotic nuclei, and mild liver hepatocyte degradation (Figure 11C). The liver sections of the group that had cyclosporine and persimmon treatment displayed a few pyknotic nuclei, a normal central vein, and modest hepatic hepatocyte degradation (Figure 11D).

#### 3.2.2. Combating Oxidative Stress in Cyclosporine-Induced Cholestasis

**Figure 12:**
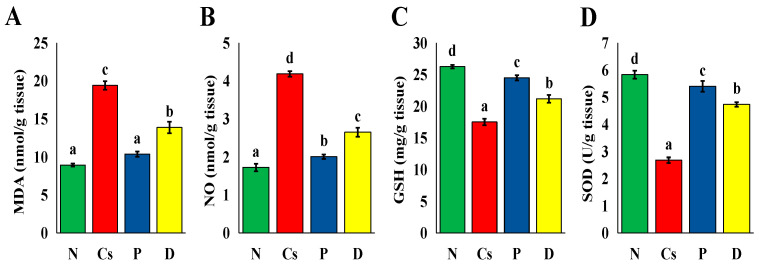
The liver oxidative markers. (**A**) MDA, (**B**) NO, (**C**) GSH, (**D**) SOD activity. The mean ± SEM (n = 6) is used to express all data. There is a substantial difference (*p* < 0.05) between mean values with distinct letters (a ˂ b ˂ c ˂ d). N stands for normal; Cs for cyclosporine; P for pomegranate; and D for persimmon.

Cyclosporine treatment caused a significant (*p* < 0.05) increase in MDA and NO levels compared to group N (Figure 12). On the other hand, the administration with pomegranate or persimmon juices, particularly pomegranate juice (*p* < 0.05), caused a significant decrease in MDA and NO levels compared to the Cs group. Additionally, cyclosporine treatment caused a significant (*p* < 0.05) decrease in GSH and SOD levels compared to group N (Figure 12). Following pomegranate and persimmon juice intervention, the P and D groups’ GSH levels significantly increased (*p* < 0.05). The GSH and SOD levels in the P group were noticeably (*p* < 0.05) greater than those in group D.

A medical disorder known as cholestasis occurs when there is a disruption in the bile’s generation, secretion, and excretion, which stops the bile from flowing normally from the liver to the intestines [76]. Through processes predominantly fueled by oxidative stress, inflammation, and cellular death, the ensuing intracellular buildup of toxic bile components damages parenchyma [77]. The two main mechanisms of cyclosporine-induced cholestasis are inflammation and oxidative stress [10]. Inflammatory cell infiltration has been shown to increase ROS production by bile duct epithelial cells, which in turn exacerbates cell damage. Antioxidant factors, such as GSH, CAT, and SOD, are the first means by which the body protects itself against the excessive production of ROS. In the cholestasis model, elevated oxidative stress indicants in the liver indicated that oxidative stress is a major contributor to cholestasis [11]. In the present study, the intervention with either pomegranate or persimmon juices exhibited amelioration in the oxidative marker. This may be attributed to the bioactive compounds in these juices. The phenolic compounds found in pomegranates, such as gallic acid, ellagic acid, anthocyanins, and flavonoids, are primarily responsible for the antioxidant activity [78]. Proanthocyanidins, tannin, carotenoids, and flavonoids are biologically active substances that decrease cell damage, prevent lipid peroxidation, and neutralize reactive oxygen species, which may be the cause of persimmon’s antioxidant activity [79]. Although the effect of both juices on the nuclear factor erythroid 2-related factor (Nrf2) pathway has not been assessed in the present study and it is required in further research, studies have indicated that the bioactive compounds in the juices, including vitamin C, may alleviate oxidative damage and inflammation via the Nrf2/Keap1 signaling pathway [80]. Additionally, gallic acid exhibits exceptional antioxidant benefits and may contribute to the suppression of cyclosporine-induced oxidative stress by stimulating Nrf2 and the activation of antioxidant genes [81].

#### 3.2.3. Suppression of Inflammation in Cyclosporine-Induced Cholestasis

**Figure 13:**
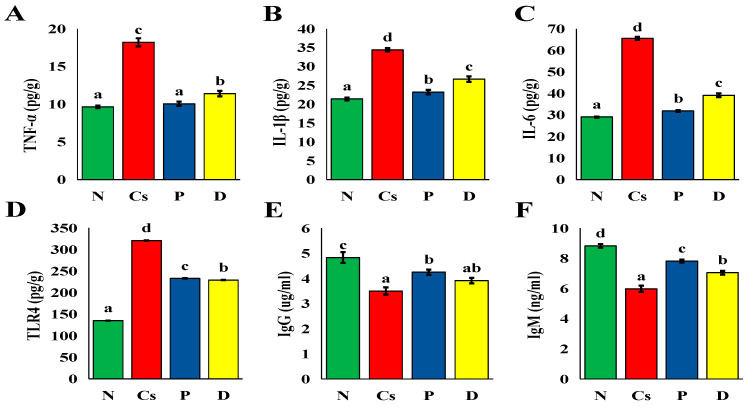
The inflammatory and immunity markers. (**A**) TNF-α, (**B**) IL-1β, (**C**) IL-6, (**D**) IgG, (**E**) IgM, (**F**) INF-γ. The mean ± SEM (n = 6) is used to express all data. There is a substantial difference (*p* < 0.05) between mean values with distinct letters (a ˂ ab ˂ b ˂ c ˂ d). N stands for normal; Cs for cyclosporine; P for pomegranate; and D for persimmon.

Cyclosporine delivery resulted in a significant (*p* < 0.05) increase in TNF-α, IL-6, IL-1β, and TLR4 levels (*p* < 0.05) compared to group N (Figure 13). Treatment with pomegranate or persimmon juices significantly reversed these changes (*p* < 0.05). Pomegranate juice outperformed persimmon in terms of its ability to lower TNF-α, IL-6, IL-1β, and TLR4 levels (*p* < 0.05). Additionally, when cyclosporine was administered, IgG and IgM levels significantly decreased (*p* < 0.05) in comparison to group N. Treatment with pomegranate or persimmon juices significantly reversed these changes (*p* < 0.05). Pomegranate juice outperformed persimmon juice in its ability to lower IgM levels (*p* < 0.05). Antibodies called immunoglobulins IgG and IgM are essential for the immune system’s reaction to inflammation and infection. IgG offers specialized, long-term protection, whereas IgM acts as the initial line of defense against novel pathogens. Active inflammation is indicated by elevated levels of either [82].

In the present study, following the cyclosporine treatment, the levels of TLR4, TNF-α, IL-6 and IL-1β increased. This may be attributed to the cholestasis induction. Neutrophils, macrophages Kupffer cells, mast cells, NK cells, and even T cells are stimulated by a variety of factors such as bile acids, inflammatory chemokines, and complement, can be activated and accumulate in the cholestatic liver, and with the involvement of inflammatory mediators and modulation by cytokines, can lead to destruction of hepatocytes and bile duct epithelial cells and exacerbate the progression of cholestatic liver disease [67]. In contrast, the intervention with pomegranate or persimmon juices resulted in a decrease in the levels of TLR4, TNF-α, IL-6 and IL-1β. This may indicate the suppression of inflammatory response in cyclosporine-induced cholestasis. The inhibition of TLR4 may be one of the anti-inflammatory mechanisms, as TLR4 is a key receptor on liver cells (Kupffer cells, stellate cells), triggering the release of inflammatory cytokines via activating downstream nuclear factor kappa B (NF-κB) signaling pathways [83]. Bacterial overgrowth and increased intestinal permeability are common outcomes of cholestasis, which enable bacterial products like LPS to enter the portal circulation and activate hepatic TLR4. Fibrosis and hepatocyte damage are encouraged by TLR4 activation. Hence, TLR4 is a possible therapeutic target since the inhibition of it can lessen liver damage [3]. From the findings of the present study, pomegranate and persimmon juices inhibited the increase in TLR4 in vivo and their major constituents (gallic acid and catechin) blocked the active pocket of TLR4 in vitro. The other abundance of polyphenols, vitamin C, tannins, carotenoids, and flavonoids in pomegranate and persimmon juices may be involved in the anti-inflammatory attributes and capacity to lower inflammatory cytokines, which aid in the diminution of oxidative stress and chronic inflammation [84,85]. Vitamin C may be involved in the suppression of inflammatory response as it has a more distinct effect on inflammatory signaling in cells, where it clearly reduces pro-inflammatory mediator expression. The NF-κB/TNF-α pathway is one of the primary known pro-inflammatory signaling pathways impacted by vitamin C [86]. Additionally, gallic acid exhibits exceptional anti-inflammatory benefits and may contribute to the suppression of cyclosporine-induced cytokine storm by stimulating the NF-κB signaling pathway that is primarily responsible for the anti-inflammatory mechanism [81]. According to Kim et al. [87], catechins may control NF-κB and TLR4 signaling pathways. Thus, catechin may be contributed in the suppression of inflammatory response following the administration of pomegranate or persimmon juices. The results of the docking assay in the present work supported the fact that gallic acid and catechin interacted with the active pocket of both TLR4 and TNF-α, which give prediction of the ability of inhibition.

The primary organ of the immune system is the liver. The liver has the largest collection of phagocytic cells in the body, which can recognize both pathogens that enter through the gut and endogenously produced antigens. The liver is an immuno-active organ that acts as a barrier between the body and the outside environment and can cause a strong and rapid immune reaction under unfavorable conditions, according to Parlar et al. [88]. Therefore, liver protection is essential. The ability of pomegranate and persimmon juice to protect the liver may be due to its anti-inflammatory and antioxidant qualities.

• Docking assay

**Figure 14:**
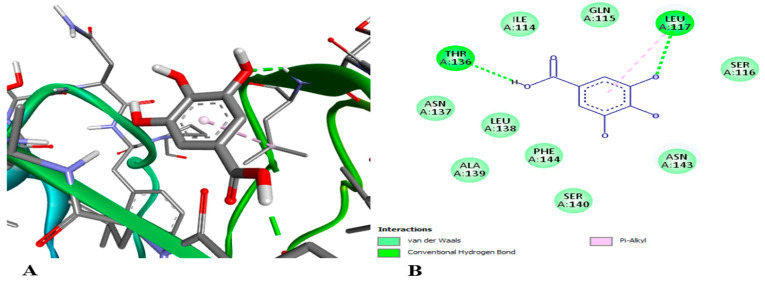
3D (**A**) and 2D (**B**) illustration of the docking and binding interactions of gallic acid in the pocket of TLR4 (pdb: 3UL7).

**Figure 15:**
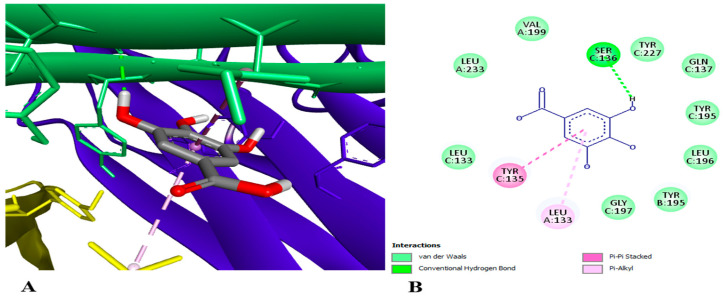
3D (**A**) and 2D (**B**) illustration of the docking and binding interactions of gallic acid in the pocket of TNF-α homotrimer (pdb: 7JRA).

**Figure 16:**
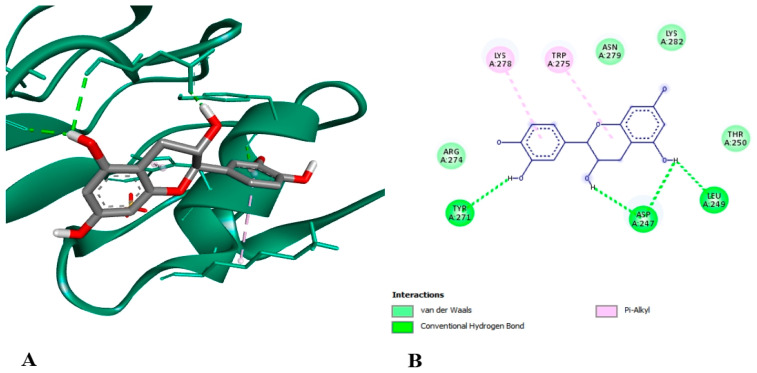
3D (**A**) and 2D (**B**) illustration of the docking and binding interactions of catechin in the pocket of TLR4 (pdb: 3UL7).

**Figure 17:**
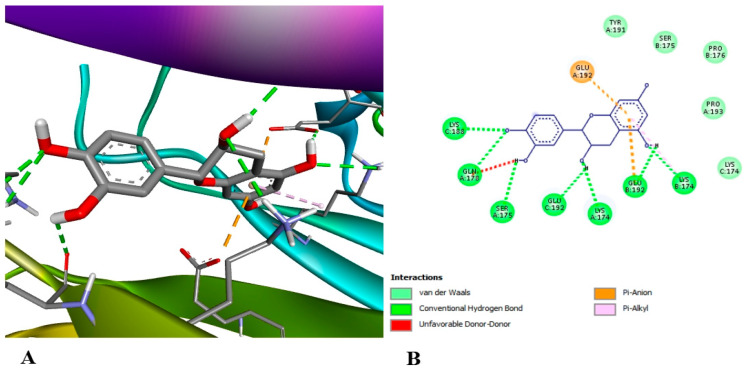
3D (**A**) and 2D (**B**) illustration of the docking and binding interactions of catechin in the pocket of TNF-α homotrimer (pdb: 7JRA).

In order to look into the possible anti-inflammatory effects of pomegranate juice or persimmon juice, human TLR4 and TNF-α were chosen as the target proteins in the docking assay. The enrichment of pomegranate or persimmon juices in gallic acid and catechin motivated us to conduct the docking assay between them and the target proteins; however, more studies on the other compounds are required. The binding affinity of catechin interactions with TLR4 and TNF-α (−6.7 and −7.6 kcal/mol, respectively) were higher than those of gallic acid with TLR4 and TNF-α (−5.6 and −4.8 kcal/mol, respectively). Figure 14 shows gallic acid linked to the active sites of TLR4 with two conventional hydrogen bonds at the THR 136 and LEU 117 residues. Figure 15 illustrates gallic acid linked to the active sites of TNF-α with conventional hydrogen bonds at SER 136 residue and hydrophobic interactions at the TYR 135 and LEU 133 residues. Figure 16 displays catechin linked to the active sites of TLR4 with three conventional hydrogen bonds at the TYR 271, ASP 247 and LEU 249 residues and two hydrophobic interactions at the TRP 275 and LYS 278 residues. Figure 17 shows catechin linked to the active sites of TNF-α with seven conventional hydrogen bonds at LYS 174 on chain A, LYS 188 on chain C, LYS 174 on chain C, GLU 192 on chain B, GLU 192 on chain C, SER 175 on chain A and GLN 178 on chain A residues. The more a compound binds to inflammatory cytokines via hydrogen and hydrophobic interactions, the greater its ability to block these cytokines and so reduce inflammation. One important trans-membrane receptor that initiates, intensifies, and maintains inflammation is toll-like receptor 4. Pro-inflammatory cytokines TNF-α, IL-1β, and IL-6 are induced when TLR4 is activated [89]. According to Zia et al. [90], TNF-α inhibitors attach to its binding sites, inhibiting receptor attachment and, consequently, the activation of downstream signaling complexes that trigger inflammation and other signaling pathways.

## 4. Conclusions

The chemical analysis of pomegranate and persimmon juices revealed the presence of bioactive compounds that have anti-inflammatory and antioxidant properties. The biochemical analysis results, metabolites, and histopathologic inspection images all indicated that pomegranate and persimmon juices have a possible hepato-protective effect against cyclosporine-induced cholestatic liver injury. Their antioxidant and anti-inflammatory properties are primarily responsible for this. These effects are likely due to the active compounds found in pomegranate and persimmon juices. The findings of the in silico assay suggested that pomegranate and persimmon juices can reduce liver inflammation by suppressing TNF-α and TLR4. However, further studies on the effect of pomegranate and persimmon juices on molecular pathways such as the nuclear receptor nuclear factor erythroid-derived 2-like 2 (Nrf2) are required. Also, molecular docking using other bioactive compounds in pomegranate and persimmon juices are required.

## Figures and Tables

**Table 1:** Organic acid content (mean ± SD) of pomegranate and persimmon juices.

	Pomegranate	Persimmon
Oxalic acid	118.52 ± 0.1 *	67.33 ± 0.05
Formic acid	1781.78 ± 0.06	ND
Acetic acid	145.21 ± 0.04	ND
Citric acid	10,272.24 ± 0.2 *	1946.67 ± 0.1
Succinic acid	219.45 ± 0.04	2699.24 ± 0.2 *
Propionic acid	642.15 ± 0.03 *	508.63 ± 0.04

The symbol * refers to the significant difference using *t*-test. ND, not detected.

**Table 2:** Detected phenolic compounds of pomegranate and persimmon juices.

	Pomegranate	Persimmon
Phenolic Compounds	Concentration (µg/g)	
Gallic acid	123.20	50.69
Chlorogenic acid	3.54	1.13
Catechin	37.78	2.67
Methyl gallate	1.36	3.80
Coffeic acid	0.44	1.91
Syringic acid	2.40	9.68
Pyro catechol	6.27	0.00
Rutin	0.00	0.98
Ellagic acid	1.47	0.75
Coumaric acid	0.19	1.77
Vanillin	1.09	0.00
Ferulic acid	1.48	0.47
Naringenin	4.35	1.68
Rosmarinic acid	0.64	2.05
Daidzein	0.00	0.21
Querectin	0.70	2.96
Cinnamic acid	0.08	0.00
Kaempferol	0.28	0.00

**Table 3:** Changes in the growth performance parameters.

	N	Cs	P	D
Initial body weight (g)	157.83 ^a^ ± 2.99	157.33 ^a^ ± 3.57	157.33 ^a^ ± 2.97	157.67 ^a^ ± 2.52
Final body weight (g)	245.17 ^b^ ± 6.37	205.83 ^a^ ± 4.00	243.17 ^b^ ± 3.82	218.83 ^a^ ± 7.92
Body weight gain (g)	87.33 ^b^ ± 5.86	48.50 ^a^ ± 5.34	85.83 ^b^ ± 4.45	61.17 ^b^ ± 6.35
Total food intake (g)	591.00 ^c^ ± 7.72	519.83 ^a^ ± 4.28	554.83 ^b^ ± 5.44	542.50 ^a^ ± 4.72
Food efficiency ratio	0.15 ^b^ ± 0.01	0.09 ^a^ ± 0.01	0.15 ^b^ ± 0.01	0.11 ^a^ ± 0.01
Liver weight (g)	6.13 ^a^ ± 0.15	8.06 ^c^ ± 0.12	6.78 ^b^ ± 0.21	6.93 ^b^ ± 0.27
Liver weight (%)	2.51 ^a^ ± 0.09	3.92 ^c^ ± 0.07	2.79 ^a^ ± 0.1	3.20 ^b^ ± 0.20

The mean ± SEM (n = 6) is used to express all data. There is a substantial difference (*p* < 0.05) between mean values with distinct letters (a ˂ b ˂ c). N stands for control normal group; Cs for cyclosporine; P for pomegranate; and D for persimmon.

**Table 4:** Profile of some metabolites (mean of area%) detected by GC-MS for blood of the studied groups.

No	Compound	Rt (min)	*p* Value	Rat Groups			
				N	Cs	P	D
1	Succinic acid	5.70	0.005	0.39	0.47	0.39	0.40
2	Propanoic acid	6.20	0.001	6.54	4.36	6.58	6.82
3	Alanine	7.05	0.001	2.10	1.61	2.16	2.17
4	Glycine	7.35	0.008	0.57	0.41	0.51	0.49
5	L-Valine,	9.47	0.006	1.15	0.94	1.18	1.04
6	L-Leucine	10.71	0.001	0.84	0.63	0.99	0.91
7	Serine	12.70	0.001	1.47	1.02	1.52	1.59
8	L-Threonine	13.29	0.001	1.9	1.23	2.00	2.00
9	L-Methionine	14.99	0.001	0.24	0.18	0.34	0.34
10	Proline	15.96	0.001	2.05	0.99	2.01	2.17
11	L-Lysine	17.81	0.001	0.37	0.21	0.39	0.41
12	L-Glutamic acid	17.92	0.002	0.21	0.16	0.22	0.25
13	L-Tyrosine	22.54	0.001	0.16	0.28	0.21	0.20
14	Tryptophan	27.83	0.001	0.43	0.67	0.51	0.45
15	Citrulline	21.62	0.001	0.42	0.14	0.42	0.3
16	Urea	10.17	0.001	9.10	14.53	10.76	11.66
17	Creatinine	16.67	0.004	0.4	0.53	0.44	0.47
18	Phosphoric acid	20.74	0.006	0.69	0.59	0.93	0.92
19	D-Fructose	19.30	0.001	0.18	0.25	0.18	0.18
20	D-Glucose	23.02	0.001	1.03	1.77	1.15	1.04
21	Cholesterol	39.09	0.001	1.53	2.66	1.04	1.15

Rt = retention time; (n = 3). N stands for normal; Cs for cyclosporine; P for pomegranate; and D for persimmon.

## Data Availability

The original contributions presented in this study are included in the article. Further inquiries can be directed to the corresponding author.
